# Factors influencing bacterial adhesion to contact lenses

**Published:** 2012-01-08

**Authors:** Debarun Dutta, Nerida Cole, Mark Willcox

**Affiliations:** 1 Brien Holden Vision Institute, Sydney, NSW, Australia; 2 Vision Cooperative Research Centre, Sydney, NSW, Australia; 3 School of Optometry and Vision Science, The University of New South Wales, Sydney NSW, Australia

## Abstract

The process of any contact lens related keratitis generally starts with the adhesion of opportunistic pathogens to contact lens surface. This article focuses on identifying the factors which have been reported to affect bacterial adhesion to contact lenses. Adhesion to lenses differs between various genera/species/strains of bacteria. *Pseudomonas aeruginosa,* which is the predominant causative organism, adheres in the highest numbers to both hydrogel and silicone hydrogel lenses in vitro. The adhesion of this strain reaches maximum numbers within 1h in most in vitro studies and a biofilm has generally formed within 24 h of cells adhering to the lens surface. Physical and chemical properties of contact lens material affect bacterial adhesion. The water content of hydroxyethylmethacrylate (HEMA)-based lenses and their iconicity affect the ability of bacteria to adhere. The higher hydrophobicity of silicone hydrogel lenses compared to HEMA-based lenses has been implicated in the higher numbers of bacteria that can adhere to their surfaces. Lens wear has different effects on bacterial adhesion, partly due to differences between wearers, responses of bacterial strains and the ability of certain tear film proteins when bound to a lens surface to kill certain types of bacteria.

## Introduction

Contact lens materials and consequently their physical properties have been modified substantially over the decades with the aim of providing clear vision with comfortable and safe lens wear. However, adhesion and colonization by microorganisms, particularly bacteria, on contact lenses continues to be implicated in several adverse events including microbial keratitis (MK) [1], contact lens related acute red eye (CLARE) [2], contact lens peripheral ulcer (CLPU) [3] and infiltrative keratitis (IK) [2]. MK is a rare but serious complication of contact lens wear may result in vision loss as a consequence of corneal scarring [4]. Depending on the study design and location, contact lens wear accounts for approximately 12% to 66% of all events of microbial keratitis [5-11]. The annualized incidence of MK ranges between 9.3 to 20.9 during overnight wear of lenses and 2.2 to 3.5 per 10,000 wearers during daily wear of lenses [12-15]. CLARE, CLPU, and IK are relatively common inflammatory complications resulting from microbial contamination of lenses with CLARE occurring in as many as 34% of patients in a study of continuously worn hydrogel lenses [16]. Continuous or extended wear is sometimes adopted because of its convenience compared to daily wear. However there is clear link between contact lens extended wear and corneal infection and inflammation [17]. Extended wear of contact lenses is one of the main risk factors for developing corneal infection [18]. Despite the suspected role of bacterial adhesion to contact lenses as the primary occurrence in many of these adverse events, the factors which are critical to determining this adhesion are still not well understood. This review examines the literature in relation to soft contact lenses (currently the most popular modality) with a focus on the factors which are known to influence adhesion. An improved understanding of this phenomenon may lead to novel strategies for the modulation of bacterial adhesion and consequently reductions in the incidences of bacterially driven adverse responses. The review will initially detail what bacterial factors are known to affect adhesion, including growth characteristics and biofilm formation. Subsequently, contact lens factors including surface chemistry and wear schedule will be examined.

## Discussion

### 

#### Bacterial characteristics

Gram negative bacteria are the predominant causative agents in contact lens-related MK, with *Pseudomonas* species being the most commonly isolated organism [12,14,19]. *Serratia marcescens,* coagulase-negative *staphylococci* and *Staphylococcus aureus* are often the next most commonly identified causative organisms [19-21]. The range of organisms associated with contact lens MK may show regional variation [12], with Gram negative bacteria being more common in tropical climates. Here we will review the characteristics of *P. aeruginosa*, *S. aureus* and coagulase-negative *Staphylococci* that affect their adhesion to contact lenses. In general, the bacterial attachment process to any surface can be divided into two stages. The first stage is one of temporary adhesion, in which bacteria can break away from the surface. This stage is largely mediated by London-Van der Waals forces [22]. In the second stage, defined by Marshall et al. [22] as one of irreversible adhesion, a time-dependent firm union occurs in which bacteria no longer exhibit Brownian motion and cannot be removed by washing. After these stages of initial adhesion, the adherent bacteria can then progress to form a biofilm which further contributes to the anchoring of the bacteria to the surface [23].

*P. aeruginosa* is a ubiquitous environmental Gram negative bacterium, with a complex genetic makeup enabling its survival in a wide variety of nutritional environments. These characteristics contribute to the mechanisms by which it adheres to contact lenses, although these are not yet fully understood. While cell surface appendages termed pili and flagella can participate in the adhesion processes of *P. aeruginosa* [24,25], non-piliated *Pseudomonas* can also adhere to contact lenses [26]. The cell surface hydrophobicity of *P. aeruginosa* also contributes to its adhesion to contact lenses [27]. The prominent adhesive nature of *P. aeruginosa* is the result of its unique physio-chemical characteristics. The strength of bacterial attachment is often influenced by their surface hydrophobicity. Organisms with greater surface hydrophobicity adhere in greater numbers than hydrophilic organisms. This phenomenon could explain the greater adhesive nature of *P. aeruginosa* than *Staphylococcus*. *P. aeruginosa* GSU#3 is reported to be highly hydrophobic with a surface water contact angle of 132° compared to that of various *S.aureus* strains which ranges from 20° to 36° [28].

Adhesion of *P. aeruginosa* varies considerably between strains [27,29-34]. *P*. *aeruginosa* strains isolated from human corneas during keratitis adhere to soft contact lenses in significantly greater numbers than isolates from other body parts. Even then there are differences between clinical isolates in their ability to adhere to surfaces [31,35]. However, *P. aeruginosa* strains can be classified as invasive and cytotoxic based on the presence of several transcriptional genes, and while one report showed no direct correlation between the cytotoxic or invasive properties and degree of their adhesion to hydrogel and silicon hydrogel lenses [29], other reports have shown that cytotoxic strains (those carrying the Exotoxin U gene) are more frequently resistant to hydrogel contact lens disinfections [36] and generally strong biofilm producers (on a polystyrene surface) [37].

Since the last decade, there have been several reports of the growing incidence of keratitis caused by microorganisms other than *P. aeruginosa* such as coagulase negative *Staphylococci* [38,39]. Some studies reported that *Staphylococcus epidermidis* accounts for as many as 45% of all cases bacterial keratitis [40,41]. Similar to *Pseudomonas*, adhesion of this organism is also reported to be strain specific [42-45]. *S. epidermidis* can adhere to a variety of different surfaces using a polysaccharide adhesin (PS/A) contained within an exopolysaccharide (slime) capsule [45]. Variation of expression of PS/A affects the degree of *S. epidermidis* adhesion to biomaterials [43,45,46]. These differences may also be related to the absence of the intracellular adhesin operon in the biofilm negative strains [47]. This operon is involved in the production of the polysaccharide intercellular adhesin (PIA) that is functionally necessary for cell-to-cell adhesion and biofilm formation [48]. The expression of exopolysaccharide and adhesins differs for each *S. epidermidis* strain leading to differences in their adhesion [49].

*Staphylococcus aureus* is another microorganism commonly isolated from MK and inflammatory events including contact lens peripheral ulcer (CLPU) [3]. Thakur et al. [50] determined that different strains of *S. aureus* adhered to a hydroxyethylmethacrylate (HEMA)-based contact lens in different numbers. However, there have been no publications on the bacterial factors that are associated with adhesion of *S. aureus* to lenses.

Overall, *P. aeruginosa* usually shows significantly greater adhesion to unworn silicone hydrogel or hydrogel lenses compared to *Staphylococcus* species [28,29,34,42-44,51] and these findings are summarized in Table 1 and Table 2. Similar findings are reported for other bacteria such as *Streptococcus pneumoniae* [44], *Hemophilus influenzae* [44], *Micrococcus luteus* [52], and *Serratia marcescens* [29] in comparison to *P. aeruginosa*. Results from Bandara et al. [44] (Table 1) show an order of magnitude higher adhesion ratio between *P. aeruginosa* and *S. epidermidis* than most other studies. This is most likely a result of the strains tested; in this case *P. aeruginosa* 6294, which shows greater level of adhesion than other *Pseudomonas* strains and *S. epidermidis* 5 which is less adhesive compared to other strains (unpublished data). Interestingly, results reported by Kodjikian et al. [42] consistently showed higher adhesion ratios between *P. aeruginosa* and *S. epidermidis* compared to results from Henriques et al. [43] to various lens materials. This is possibly due to the nutritionally limiting phosphate buffered saline (PBS) used by Kodjikian et al. [42] compared to nutritionally rich artificial tears used by Henriques et al. [43] In nutritionally poor media *S. epidermidis* can show reduced cell viability especially if incubated for longer periods of time. Similarly, Borazjani et al. [29] (Table 2), using PBS as media for both bacteria, showed a very high comparative adhesion ratio between *P. aeruginosa* and *S. aureus*. The comparable adhesion between these two species in data from Zang et al. [52], Willcox et al. [53], and Vermeltfoort et al. [54] (Table 2) under similar assay conditions appears to be mainly strain driven, but still showing higher adhesion by *P.aeruginosa*.

**Table 1:** Comparison of bacterial adhesion levels to various lens materials for *P. aeruginosa* and *S. epidermidis*.

**Ratio of *P. aeruginosa* adhesion compared to *S. epidermidis***	**Lens materials**	**References**
16 - 45	Etafilcon A	[42]
533 - 717	Etafilcon A	[44]
5–11	Etafilcon A	[43]
2–3	Polymacon	[57]
72 - 78	Balafilcon A	[42]
1–5	Balafilcon A	[43]
1–3	Galyfilcon A	[43]
34 - 125	Galyfilcon A	[42]
22 - 32	Lotrafilcon B	[42]
1–5	Lotrafilcon A	[43]

**Table 2:** Comparison of bacterial adhesion levels to various lens materials for *P. aeruginosa* and *S. aureus*.

**Ratio of *P. aeruginosa* adhesion compared to *S. aureus***	**Lens materials**	**References**
0.7	Etafilcon A	[52]
26 - 85	Etafilcon A	[89]
0.9	Etafilcon A	[53]
185	Etafilcon A	[29]
28	Balafilcon A	[54]
28	Balafilcon A	[29]
41 - 47	Balafilcon A	[89]
0.9	Lotrafilcon A	[54]
44 - 51	Lotrafilcon B	[89]
37 - 65	Senofilcon A	[89]
7 - 17	undisclosed	[28]

In most of the studies PBS has been used as bacterial suspension media [29,31,32,42,51,55-57]. This buffer solution helps to maintain a constant pH and osmolarity. Highest bacterial adhesion with PBS has been observed at pH 7 [42]. Some studies have used instead sterile saline [52,58] but as both PBS and saline are nutritionally inert and thus longer incubation with PBS and saline may underestimate total cell numbers (especially for the more fastidious microbes such as Staphylococci which may die upon prolonged exposure). Some studies have used a complex and nutritionally rich media such as trypticase soya broth as bacterial suspension media, while other studies have used dilutions of this media in PBS [44,59]. In an attempt to more closely replicate adhesion that may occur during lens wear, several other studies have used artificial tears as the bacterial suspension fluid [43,60]. Any differences seen between adhesion levels between studies using these different media might be related to affects of electrolyte concentration and ionic charge of suspending media, as well as the nutritional fastidiousness of the bacteria. Furthermore, nitrogen or carbon limitation which can alter the ability of *P. aeruginosa* to adhere to Etafilcon A lenses [61].

Interestingly, George et al. [51] observed that the presence of *S. epidermidis* on hydrogel lens surfaces significantly reduces the adhesion of *P. aeruginosa*, but the presence of *P. aeruginosa* does not largely alter the adhesion of *S. epidermidis* [51]. This phenomenon is yet to be understood though it further supports the general premise that the normal ocular microbiota (of which coagulase-negative staphylococci such as *S. epidermidis* are predominant members) may be protective to eye. The strength of adhesion to contact lenses is also strain dependant. A rinsing step after bacteria adhesion can remove roughly 90% more of *S. epidermidis* compared to *P. aeruginosa* and passage through a air/liquid interface can detach more *S. aureus* than *P. aeruginosa* [42,51]. In contrast, a recent investigation of adhesion forces between bacteria and contact lenses, lens storage cases or the cornea (measured using atomic force microscopy) suggested that staphylococci and *Serratia liquifaciens* adhered significantly stronger than *P. aeruginosa,* and this might result in slightly higher transmission rates of *P. aeruginosa* to the cornea [62].

Using five different *P. aeruginosa* strains, Williams et al. [31] sought to determine whether an increasing bacterial concentration (1×10^7^ to 1×10^9^) results in greater viable bacterial counts on contact lenses. Maximum adhesion was seen when 1×10^9^ CFU/ml bacteria were added to lenses, except in the case of *P. aeruginosa* 6294, which reached maximum adhesion to worn lenses at 1×10^8^ CFU/ml. Interestingly George et al. [51] found that re-exposure of *P. aeruginosa* to fresh lenses after the bacterial inoculum had been previously allowed to adhere to a contact lens resulted in lower adhesion than to the initial lens [51]. This phenomenon suggests a limited number of bacterial cells in a standard inoculum are responsible for adhesion, suggesting a certain phenotype of bacterial cells within a population (approximately 10% of the cells) is responsible for most of the adhesion seen. This could be due to the use of cells grown in static culture to stationary phase, where cells of differing phenotype could be present. Indeed, Williams et al. [31], found that *P. aeruginosa* strains grown to stationary phase adhered in higher levels that those grown to exponential phase.

The time required for irreversible bacterial attachment and biofilm formation on the lens surface is a crucial factor that can differ between bacterial types. The adhesion of *S. epidermidis* on hydrogel lenses tends to be slowly incremental over 2 h while *P. aeruginosa* adhesion is as quick as 5 min [51]. Miller et al. [35] reported adhesion of *P. aeruginosa* nonmucoid isolate number 3 increased with time, peaking after 3 h and then remaining constant. Duran et al. [56] reported a steady increase in adhesion of an MK isolate of *P. aeruginosa* to Polymacon and Lidofilcon A lenses from 2 min to 1 h. Subsequent studies by Stapleton et al. [59] supported these data and showed rapid attachment up to 10^7^ cells per lens, and adhesion reached maximum after 45 min. Glycocalyx formation (i.e., biofilm formation) occurred after 30 min incubation with a bacterial inoculum of 10^7^ organisms per ml [59]. Andrews et al. [63], using an ATP based bioluminescent assay and image analysis, reported that the adhesion of *P. aeruginosa*, *S*. *epidermidis*, and *S. marcescens* was maximal at 4–6 h but this was followed by a metabolic decline after 18 h [63]. The decrease in metabolic activity is characteristic of a biofilm mode of growth [64]. However, others have shown that viable cell numbers on several lens materials (hydrogel and silicone hydrogel) significantly increased up to 16 to 24 h after incubation [33,60]. Randler et al. [60] noticed a decrease in viable bacterial numbers on silicone hydrogel lenses exposed to an artificial tear fluid within a few hours. This observed decrease may be due to the antimicrobial components such as lysozyme in the artificial tear fluid.

Overall the main bacterial factors that influence adhesion are their cell surface hydrophobicity, use of different strains, and the suspension media. Most studies show that the adhesion of *P. aeruginosa* is higher than other bacterial types to most lens types, perhaps being one reason for the predominance of this bacterium in microbial keratitis. Adhesion of *P. aeruginosa* to lenses is rapid, usually occurring within 1 h, and biofilm formation can occur with 24 h of initial adhesion.

#### Contact lens material characteristics

Since the introduction of soft contact lenses to the contact lens market by Bausch and Lomb in 1971, exhaustive research has been conducted to investigate and improve soft lens materials. Susceptibility of this and all biomaterials to bacterial colonization has been a major concern of manufacturers, researchers and practitioners. Low oxygen transmissibility of hydrogel lenses was overcome by the introduction of silicone hydrogel lenses in 1999. These lenses have successfully reduced various clinical signs of corneal hypoxia. It was anticipated that contact lens related infiltrative events would also be reduced by these lenses but this is not evident [65]. Bacterial adhesion to lens surfaces is heavily influenced by lens material characteristics. The following are the distinguishing lens material characteristics and their effect on bacterial adhesion.

Several authors have observed greater levels of adhesion of various strains of *P. aeruginosa* to lenses composed of non-ionic polymers compared to those with ionic polymers [35,59]. Initial adhesion of *S. aureus* was higher to an ionic hydrogel compared to a non-ionic hydrogel [66]. The Federal Drug Administration (FDA) classifies soft contact lens materials as being of high water content (more than 50%) or low water content (less than 50%). Results of research into bacterial adhesion based on water content have been remarkably consistent with multiple studies concluding that bacterial adhesion increases inversely to the water content [30,34,35,42,46,67]. Garcia-Saenz et al. [46] showed increased adhesion to low water content hydrogel lenses of two strains of *S. epidermidis.* Kodjikian et al. [42], Miller et al. [30,35], and Cook et al. [67,68] found the same effect for strains of *P. aeruginosa*, although Miller et al. [35] reported no strict correlation between water content and adherence. However, two studies could not establish any relation between water content and adhesion [69,70] and there appears to be no relationship between the numbers of bacteria isolated from lenses after wear and water content [70]. Silicone hydrogel lenses are all of relatively low water content and so water content is likely to be a minor factor in the bacterial adhesion to this lens type. Polymer composition and surface hydrophobicity can mask the effects of water content, reducing the influence of this characteristic on the degree of bacterial adhesion [35].

Hydrophobicity is a crucial contact lens surface property. Common adhesive patterns of most bacterial isolates to various substrata indicate that hydrophobic surfaces attract greater numbers of bacteria than hydrophilic surfaces [58,71-74]. Bacteria attach with higher affinity to low-energy, hydrophobic surfaces than to high energy, hydrophilic surfaces [71]. Hydrophobicity of silicone hydrogels such as Lotrafilcon A, Balafilcon A, or Lotrafilcon B has been reported to be higher compared to hydrogel lenses such as Etafilcon A. In vitro *P.aeruginosa, S.aureus*, or *S. epidermidis* adhere in greater numbers to the hydrophobic silicone hydrogel lenses compared to hydrophilic hydrogel lenses [28,43,51,74]. Also, Lotrafilcon A and Balafilcon A are often reported as the most hydrophobic silicone hydrogels lenses and more bacteria adhere to these compared other silicone hydrogels [43]. It is likely that hydrophobic bacteria prefer adhering to hydrophobic lenses whereas hydrophilic bacteria adhere well to hydrophilic lenses [54]. However, Santos et al. [75] found the same amount of *S. epidermidis* adhesion to unworn silicone hydrogel and hydrogel lenses.

Detailed information regarding contact lens topography and roughness can be determined by atomic force microscopy. Giraldez et al. [58] reported that Comfilcon A and Omafilcon A lens materials had relatively smooth surfaces compared to Senofilcon A, Nelfilcon A, and Ocufilcon B. *S. epidermidis* showed stronger adhesion to the materials with higher surface roughness [58]. Similarly, with *S. epidermidis,* when adhesion to surfaces with similar hydrophobicity but differing surface roughness was examined, a higher surface roughness resulted in an increase in bacterial adhesion [76]. There are no reports investigating a link between adhesion of *P. aeruginosa* and lens surface roughness.

Contact lens wear can have significant effects on the surface properties of lenses, due to the deposition of tear film components (and possibly components of multipurpose disinfection solutions) during wear. After wear silicone hydrogel lenses show reduced surface hydrophobicity [54,75,77]. Worn hydrogel and silicone hydrogel lenses usually exhibit higher degrees of roughness than their unworn counterparts [75,78,79]. Therefore, these changes in surface characteristics of lenses during/after wear may influence bacterial adhesion. Worn Galyfilcon A and Lotrafilcon A adhere more *S. epidermidis* than unworn lenses [75]. The presence of sorbed protein can increase adhesion of *S. epidermidis* by 45% [67]. *P. aeruginosa* colonize the surface of worn extended wear contact lenses in direct proportion to lens surface deposits, preferentially adhering on areas of lens deposits [80]. In vitro *Pseudomonas* adhesion is highly correlated with the number of large (more than 150 µm) focal deposits on the lens after wear [81]. In contrast, though enzymatic cleaning is recommended for protein deposition, it does not appear to significantly reduce the adhesion of *P. aeruginosa* to lens surfaces [80].

Borazjani et al. [29] and Boles et al. [55] reported that 1 week extended wear of Balafilcon A and Etafilcon A had no major effect on the adhesion of *P. aeruginosa.* However, continuous wear of lotrafilcon A lenses reduced adhesion of the hydrophilic *S. aureus* strain 835, whereas continuous wear of balafilcon A lens significantly increased adhesion of this same strain [54]. Adhesion of the hydrophobic *P. aeruginosa* strain #3 to lenses after continuous wear was generally less than to unworn lenses, regardless of the type of lens [54]. Interestingly, *P. aeruginosa* 6294 adhered to a greater extent to the unworn Etafilcon A lenses than to 30 nights continuously worn lenses [82]. In vitro adhesion of *P. aeruginosa* significantly varies when lenses are worn by different individuals [30]. Butrus et al. [83] demonstrated that *P. aeruginosa* adhere in greater numbers to worn extended wear soft contact lenses compared to unworn lenses. In another study, worn Balafilcon A lenses have been shown to increase bacterial adhesion [84]. These differences in the effect of lens wear on adhesion may be related to bacterial strain differences, how the adhesion was measured (examining live bacteria (CFU/lens) or total bacterial cells) or to the difference between individuals [29].

It may be that it is the presence of specific adsorbed tear products on worn contact lenses that affect bacterial adhesion. Stern et al. [85] and Miller et al. [35] reported that adsorbed mucin, IgA, BSA, lysozyme, and lactoferrin enhanced the adhesion of *P. aeruginosa* GSU#3 to contact lenses. Adhesion of *P. aeruginosa* strain RT-1, a corneal ulcer isolate, was increased when albumin was adsorbed to a lens surface [86]. However, Williams et al. [31,32] and Lakshman et al. [87] showed that the presence of lactoferrin increased the total numbers of *P. aeruginosa* adhering to lenses but reduced their viability, killing the attached bacteria. In vitro, the presence of lysozyme on a lens surface has a variable impact on adhesion of *P. aeruginosa* [31,50] and *S. aureus* [52] but markedly reduces the viability of *Micrococcus luteus* [52]. The number of viable cells of *S. aureus* that adhere to contact lenses is reduced if those lenses are coated with secretory phospholipase A2 [87]. The ability of the tear film lipids cholesterol or phospholipids to modulate bacterial adhesion has been measured [88]. The lipids once adsorbed to lenses appear to have no effect on bacterial adhesion.

Overall, it is the lens surface hydrophobicity and roughness, as well as polymer characteristics such as water content and iconicity (at least for HEMA-based lenses) that appear to modulate bacterial adhesion. The effect of lens wear is not constant and may be affected by the individual who has worn a lens, and the types of proteins deposited on the lens surface.

#### Concluding remarks

It is important to note that reported adhesion rates are sensitive to study methodology and every study has its unique features. Despite this, it is still instructive to attempt some comparisons. Most of the ocular pathogens showed in vitro variation in adhesion between species and strains. *P. aeruginosa* can adhere to contact lenses the most of any bacteria test thus far, and this may be a reason it is the most predominant microorganism that causes contact lens-associated MK.
